# A Cattle Disease

**Published:** 1895-05

**Authors:** N. S. Mayo


### CORRESPONDENCE.

### A CATTLE DISEASE.

At the request of the editor I gladly furnish a concise statement of the reported contagious pleuro-pneumonia in Kansas.

At the request of Governor Morrill and with Dr. Pritchard, State Veterinarian, we investigated a disease of cattle in Morris, Geary, and Wabaunsee counties, and found them affected with a chronic pleuro-pneumonia, apparently infectious, being scattered through a number of herds. A number of cattle had died, and many more were sick. As lung diseases of cattle are extremely rare in this climate and altitude, and no similar disease ever having been known here, the general symptoms and cause of the disease (running from two to three months and more) resembling contagious pleuro-pneumonia, it was decided to be sufficiently serious to warrant a thorough investigation by an expert familiar with contagious pleuro-pneumonia. Dr. Pritchard advised the Live Stock Sanitary Commission to quarantine the affected herds, which was done. I reported the condition of the cattle to Governor Morrill, and it was decided to call on the Bureau of Animal Industry for an expert to investigate. At the Governor's request I dictated a letter to Dr. Salmon, stating the condition of the cattle, and that the disease resembled contagious pleuro-pneumonia. It was decided to keep the matter quiet until it could be thoroughly investigated; hence a letter was sent in place of a telegram for fear the press might get hold of it. In the course of an hour or so telegrams inquiring about the disease came to the Governor from Chicago and Kansas City, and the press got hold of the news through some source unknown to me.

Neither Dr. Pritchard nor myself stated that the disease was contagious pleuro-pneumonia, and the reports that I said that the disease was contagions pleuro-pneumonia, and that the State was flooded with it, are false.

Dr. Devoe, of the Bureau of Animal Industry, decided positively that the disease is not contagious pleuro-pneumonia. Dr. Devoe thinks the disease is caused by dirty corn-fodder; but I am of the opinion that a lack of water and a very poor quality is a more satisfactory explanation, as cases of the disease occurred while the animals were on green oats the last of August and early September.

The disease affects cows more seriously than younger cattle.

Experiments are being made to determine whether the disease is infectious or not, and later I shall make a more detailed report.

N. S. Mayo, M.S., D.V.S.

---

An Australian sheepbreeder is endeavoring, through breeding, to establish a family of black sheep, using only black ewes with a black ram, rejecting all that are not true to color. Since black absorbs more heat, such sheep it is thought would be better adapted to the colder climates.

